# Nonspecific protection of heat-inactivated *Mycobacterium bovis* against *Salmonella* Choleraesuis infection in pigs

**DOI:** 10.1186/s13567-022-01047-8

**Published:** 2022-04-18

**Authors:** Rita Vaz-Rodrigues, Elisa Ferreras-Colino, María Ugarte-Ruíz, Michele Pesciaroli, Jobin Thomas, Teresa García-Seco, Iker A. Sevilla, Marta Pérez-Sancho, Rafael Mateo, Lucas Domínguez, Christian Gortazar, María A. Risalde

**Affiliations:** 1 SaBio (Health and Biotechnology), Instituto de Investigación en Recursos Cinegéticos IREC (UCLM-CSIC), Ciudad Real, Spain; 2 VISAVET Health Surveillance Center, Universidad Complutense de Madrid, Madrid, Spain; 3 Istituto Zooprofilattico Sperimentale Della Lombardia E Dell’Emilia Romagna, Brescia, Italy; 4 Indian Council of Agricultural Research (ICAR), New Delhi, India; 5 Animal Husbandry Department, Thiruvananthapuram, Kerala India; 6 Animal Health Department, NEIKER-Instituto Vasco de Investigación y Desarrollo Agrario, Derio, Bizkaia Spain; 7 Department of Animal Health, Faculty of Veterinary, Universidad Complutense de Madrid, Madrid, Spain; 8 Grupo de Investigación en Sanidad Animal y Zoonosis (GISAZ), Departamento de Anatomía y Anatomía Patológica Comparadas y Toxicología, Facultad de Veterinaria, Universidad de Córdoba (UCO), Córdoba, Spain; 9 Infectious Diseases Unit, Clinical Virology and Zoonosis Group, Maimónides Biomedical Research Institute of Cordoba (IMIBIC), Reina Sofía University Hospital, Cordoba, Spain; 10 CIBERINFEC, ISCIII - CIBER de Enfermedades Infecciosas, Instituto de Salud Carlos III, Cordoba, Spain

**Keywords:** Heat-inactivated *Mycobacterium bovis*, pig, *Salmonella* Choleraesuis, trained immunity

## Abstract

Trained immunity is the capacity of innate immune cells to produce an improved response against a secondary infection after a previous unrelated infection. Salmonellosis represents a public health issue and affects the pig farming industry. In general, vaccination against salmonellosis is still facing problems regarding the control of distinct serovars. Therefore, we hypothesized that an immunostimulant based on heat inactivated *Mycobacterium bovis* (HIMB) could have an immune training effect in pigs challenged with *Salmonella enterica* serovar Choleraesuis (*S*. Choleraesuis) and decided to explore the amplitude of this non-specific immune response. For this purpose, twenty-four 10 days-old female piglets were randomly separated in three groups: immunized group (*n* = 10) received orally two doses of HIMB prior to the intratracheal *S*. Choleraesuis-challenge, positive control group (*n* = 9) that was only challenged with *S*. Choleraesuis, and negative control group (*n* = 5) that was neither immunized nor infected. All individuals were necropsied 21 days post-challenge. HIMB improved weight gain and reduced respiratory symptoms and pulmonary lesions caused by *S.* Choleraesuis in pigs. Pigs immunized with HIMB showed higher cytokine production, especially of serum TNFα and lung CCL28, an important mediator of mucosal trained immunity. Moreover, immunized pigs showed lower levels of the biomarker of lipid oxidation malondialdehyde and higher activity of the antioxidant enzyme superoxide dismutase than untreated challenged pigs. However, the excretion and tissue colonization of *S*. Choleraesuis remained unaffected. This proof-of-concept study suggests beneficial clinical, pathological, and heterologous immunological effects against bacterial pathogens within the concept of trained immunity, opening avenues for further research.

## Introduction

Traditional vaccination targets specific pathogens, relying on the adaptive immune response mediated by T and B lymphocytes [1, 2]. Nevertheless, a growing body of evidence suggests that the innate immune response is also enhanced after stimulation with certain antigens, partially protecting against infection with the same pathogen or a different one [3]. Here lies the concept of trained immunity that does not imply a high level of specificity nor amplification as it happens with adaptive immunity and which involves innate immune cells such as macrophages (MΦs) and natural killer (NK) cells [4, 5], as well as proinflammatory cytokines such as interleukin (IL)-1α and tumour necrosis factor α (TNFα) or reactive oxygen species (ROS) [6] as mediators. It seems that trained immunity relies on epigenetic and metabolic reprogramming for enhancing the functional state of innate immune cells, cytokine production and sustaining cellular memory [7–9], which could be responsible for the nonspecific protective effects produced by some immunostimulants [10, 11].

One of the most used vaccines as a model to study trained immunity mechanisms is Bacillus Calmette-Guérin (BCG), due to its ability to induce nonspecific cross-protection against a vast number of pathogens. In murine models, heterologous BCG stimulation has shown to reduce the acquisition of infections caused by *Candida albicans*, *Listeria monocytogenes*, *Salmonella typhimurium*, *Staphylococcus aureus*, *Schistosoma mansoni*, *Plasmodium* sp. and *Babesia* sp. [6, 12, 13]. Moreover, various studies revealed that infant immunization with BCG reduced the incidence of acute lower respiratory infections [14, 15]. However, oral administration of this live attenuated vaccine led to BCG shedding and detection in lymphoid organs several days postimmunization [16, 17]. To prevent this, the use of inactivated immunostimulants seems promising, since it decreases the possibility of vaccine strain spreading and makes deployment logistics easier [18].

An immunostimulant based on heat-inactivated *Mycobacterium bovis* (HIMB) was developed in 2011 for use in pigs and wild boar (*Sus scrofa*) against tuberculosis, with similar results as oral BCG in the reduction of lesions and mycobacterial culture scores [19]. Studies using HIMB in different species and applying various routes of administration proved its homologous effect against *Mycobacterium bovis* (*M. bovis*) [20–23]. In this regard, it has been demonstrated that killed mycobacteria activate a training effect in the lytic phase of phagocytosis in MΦs [24], as well as an increase in pro-inflammatory cytokines and the complement component 3 (C3) in immunized animals [25–27]. The immunological mechanisms triggered by HIMB stimulation could also favour nonspecific protection against unrelated pathogens compatible with the concept of trained immunity. However, to the best of our knowledge, there are no studies assessing the heterologous protection capacity of inactivated mycobacteria, in other words, experiments using infectious agents other than mycobacteria for challenge.

*Salmonella enterica* subspecies *enterica* serotype Choleraesuis (*S*. Choleraesuis), is a zoonotic pathogen that causes pneumonia and septicaemia in swine and can lead to miscarriage in sows [28, 29]. Aside from taking a toll on the pig farming industry, leading to major economic losses due to reductions in daily weight gain and therapy expenses [30], it also represents a threat to public health [31]. Furthermore, the prevalence of this serovar amongst its reservoir, the wild boar, is increasing in Europe [32, 33] with outbreaks in Italy [34] and Spain [35]. This serovar was also reported in Danish pig herds [36] and in weaned piglets from Serbia [37]. The increasingly common resistance to conventional antimicrobial agents and the limited capacity of vaccines to control distinct *Salmonella* serovars creates the need to explore trained immunity activators (immunostimulants) as an alternative or complementary control tool [38, 39].

The objective of this study was to evaluate if an immunostimulant based on heat inactivated *M. bovis* (HIMB) elicits a protective effect in pigs challenged with *S*. Choleraesuis. We explore the amplitude and efficacy of this non-specific immunological stimulation, a process that falls within the trained immunity concept. We hypothesize that HIMB stimulates the innate immune system and may protect pigs against *S*. Choleraesuis challenge.

## Materials and methods

### Animals and experimental design

Twenty-four 10 days-old Landrace x Large White hybrid female piglets with homogeneous weights were obtained from a pig production farm for experimental animals. They were in good health, free of clinical signs of enteric disease and seronegative to *Salmonella* species. Also, *Salmonella* spp. was not isolated from their mother’s stools. Seven days prior to start the study, they were housed in class III biocontainment animal facilities (BSL-3) situated at VISAVET Health Surveillance Centre (Madrid, Spain) for acclimatization. All animals received continuous access to water, nonmedicated pig feed and veterinary care. These individuals were randomly assigned to the experimental groups, identified via microchip and ear tag and housed in separated rooms as follows: the immunized group (*n* = 10) received two oral doses (with an interval of 3 weeks) of HIMB prior to the intratracheal *S*. Choleraesuis-challenge, the positive control group (*n* = 9) that was only challenged with *S*. Choleraesuis, and the negative control group (*n* = 5) that was neither immunized nor infected. The challenge was done using an 18G needle after sedation with xylazine (Xilagesic 2%; Laboratories Calier, Barcelona, Spain).

Animals were handled several days during the experiment for stool sample collection (SS), blood withdrawal (BW), body temperature (BT) and weight (W) measurements. They were also monitored for the appearance of clinical signs (CS) (Table 1). The stool samples were processed by microbiological culture to evaluate faecal shedding. The whole blood collected served for determining the activity of glutathione peroxidase (GPx, EC 1.11.1.9) and superoxide dismutase (SOD, EC 1.15.1.1), while malondialdehyde (MDA) levels were analysed via plasma samples. The serum obtained was used for determining pig complement C3, cytokine (IL-1β, IL-10 and TNFα) concentration and antibody titers against *Mycobacterium tuberculosis* complex (MTC).

**Table 1:** **Data collection calendar indicating the days each measure or monitorization was made**

	Days *Salmonella* Choleraesuis post-infection (dpi)									
	0	1	2	3	7	9	14	15	20	21
BT	✔	✔	✔	✔	✔		✔	✔	✔	✔
W	✔				✔		✔			✔
BW	✔			✔	✔		✔			✔
SS	✔				✔		✔			✔
CS	✔	✔	✔		✔	✔				✔

Days post-infection (dpi) that animals were handled for measurement of body temperature (BT), weight (W), blood withdrawal (BW), collection of stool samples (SS) and clinical sign (CS) monitoring.

All piglets were euthanized at 21 days post-infection (dpi) by captive bolt after sedation with xylazine (Xilagesic 2%, Laboratories Calier, Barcelona, Spain). Later, they were subjected to necropsy to assess the presence and extension of *S*. Choleraesuis macroscopic lesions in several organs (Table 2). The evaluation of these findings was performed using a lesion score: absent = 0, mild = 1, moderate = 2 and severe = 3.

**Table 2:** **Macroscopic lesions per organ taken into account for scoring**

Organ	Macroscopic lesions
*Lymph nodes*	Lymphadenomegaly and congestion
*Lung*	Interstitial pneumonia
*Heart*	Hydropericardium
*Liver*	Focal necrosis
*Spleen*	Splenomegaly and hyperaemia
*Small intestine*	Enteritis, congestion and petechiae
*Kidney*	Focal necrosis and petechiae

We collected samples from the following tissues: palatine tonsil, tracheobronchial lymph nodes (LNs), spleen and lung (cranial and caudal lobes). Samples from the lymphoid organs (palatine tonsil, tracheobronchial LNs and spleen) were cultured to evaluate tissue colonization. Lung and tracheobronchial LNs samples were fixed in 10% neutral buffered formalin, embedded in paraffin wax and routinely processed for histopathology. Furthermore, other lung tissue samples were also stored at −80 °C with RNAlater Stabilization Solution (Thermo Scientific, Wilmington, USA) for molecular studies.

### HIMB immunostimulant

The oral immunostimulant used consisted of 2 mL of sterile PBS containing approximately 10^7^ heat inactivated CFU/mL of a *M. bovis* field isolate (strain 1403; spoligotype SB0339) that had been obtained from a naturally infected wild boar. The vaccine preparation followed the protocol described by Garrido et al. [19], apart from an extended inactivation step at 83 °C for 45 min. Bacterial concentration of HIMB was determined prior to inactivation by measuring the turbidity of the suspension in MacFarland scale using a VITEK® DensiCHEK® (BioMerieux) and by plating a serially diluted aliquot onto agar-solidified Middlebrook 7H9 with glycerol (0.2% v/v) and OADC (10% v/v) (Becton Dickinson, Franklin Lakes, NJ, USA).

### Challenge

The *S*. Choleraesuis variant Kunzendorf used as challenge strain (DICM15/00069, VISAVET reference) was isolated from the spleen of a naturally infected wild boar and cultivated in a Luria–Bertani broth at 37 °C for 24 h, following the protocol described by Ibrahim et al. [40]. Each dose consisted of 10^6^ CFU *S*. Choleraesuis in one mL.

### Microbiological culture and *Salmonella* excretion

To analyse faecal shedding and bacteria tissue distribution the semiquantitative microbial culture method based on ISO 6579:2002/AMD 1:2007 was applied but by performing ten-fold serial dilutions to enable the estimation of bacterial concentrations in each sample [41]. Once known the last dilution where bacterial growth occurs, the number of CFU/gram per sample was calculated.

### Histopathology

A standard procedure for haematoxylin and eosin (HE) staining was used for microscopical examination of lung and tracheobronchial LNs. Although tissues of all animals were processed for histopathology, only samples from immunized and control positive groups were analysed. The main lesions caused by *S*. Choleraesuis in these organs [42–44], were evaluated giving a lesion score based on the severity and distribution of the lesions in each individual, where absent = 0, mild = 1, moderate = 2 and severe = 3. The histopathological lesions considered for lung scoring were oedema (alveolar or interstitial), congestion, haemorrhages, hemosiderosis, inflammatory infiltrate (mononuclear, polymorphonuclear [PMN] or mixed), bronchial exudate, hyperplasia of seromucous glands and of epithelial cells, and necrosis. As for the tracheobronchial LNs the following lesions were considered: oedema, congestion, haemorrhages, hemosiderosis, lymphoid depletion, and necrosis.

### Oxidative stress biomarkers (MDA, GPx and SOD)

MDA plasma levels were measured using high-performance liquid chromatography (HPLC), as previously described by Agarwal and Chase [45]. Concentrations were expressed as mmol MDA/L of plasma samples and estimated by applying the linear regression equation of the standard curve to the unknown sample peak-area. The activities of GPx and SOD were determined spectrophotometrically using the Ransel and Ransod kits (Randox Laboratories, Crumlin, UK), respectively, following the instructions from the manufacturer. The results of the GPx and SOD activity were expressed in µmol/L of whole blood.

### Analysis of complement C3 in serum

Pig serum C3 concentration was measured using a commercial sandwich ELISA kit (Cusabio Technology LLC, Houston, USA), following the manufacturer’s guidelines. A standard curve was generated alongside with a regression analysis to determine each sample C3 concentration in µg/mL.

### Serum cytokines (IL-1β, IL-10 and TNFα)

The serum concentrations of the proinflammatory cytokines IL-1β, IL-10 and TNFα were examined via specific swine commercial sandwich ELISA Kits (Invitrogen, Waltham, USA), following the instructions from the manufacturer. A standard curve was run per assay, allowing the determination of each cytokine concentration in pg/mL.

### Antibody titers against MTC

Serum samples were analyzed by ELISA to detect antibodies against MTC in all the groups studied at the different blood collection time points. Bovine tuberculin purified protein derivative (bPPD) (CZ Veterinaria SA, Porriño, Spain) and an immunopurified subcomplex protein from bPPD, named P22 [46], were used as antigens in an indirect in-house ELISA previously described by Thomas et al. [47]. The estimated sensitivity and specificity of this ELISA in swine was 77.3% and 97.3% for PPDb, as well as 84.1% and 98.4% for P22, respectively [47].

### Assay of pulmonary cytokines by real time qPCR

Lung tissue samples were used to extract mRNA. Firstly, 15 mg of tissue was disrupted with a scalpel and homogenized using a needle and syringe. Secondly, total mRNA was isolated using Rneasy Plus Mini Kit (Qiagen, Hilden, Germany), following manufacturer’s instructions.

The reverse transcription of total mRNA into cDNA was performed using the iScript cDNA Synthesis Kit (Bio-Rad, Hercules, USA), following the guidelines of the manufacturer. We used a total volume reaction of 20 µL containing: 2 µL of sample mRNA, 1 µL of iScript Reverse Transcriptase, 4 µL of 5× iScript Reaction Mix and 13 µL of nuclease-free water. The complete reaction mix was incubated in a thermal cycler for priming during 5 min at 25 °C, then the reverse transcription was carried out for 20 min at 46 °C followed by inactivation at 95 °C for 1 min.

Both concentration (ng/µL) and purity of mRNA and cDNA samples were assessed by quantification of the nucleic acids at an optical density of 260 nm (OD260) and the ratio of absorbance at 260/280 nm, using a Nanodrop One spectrophotometer (Thermo Scientific, Waltham, USA). At the end, concentrations were standardized at 20 ng/µL for mRNA and 200 ng/µL for cDNA. Afterwards, all samples were stored at −80 °C.

The amplification of the synthesized cDNA was realized using the CFX96 real-time PCR detection system (Bio-Rad, Hercules, USA) and the quantification was performed by utilizing SYBR green chemistry (Power SYBR Green, Applied Biosystems, Waltham, USA). All real time PCR reactions were carried through in a 96 well plates (Applied Biosystems, Waltham, USA). The concentration selected for primer working solution was 10 μM. For a total volume of 20 µL, the PCR mixture contained: 10 µL of SYBR Green Master Mix (Bio-Rad, Applied Biosystems, Waltham, USA), 1 µL of primer forward, 1 µL of primer reverse, 2 µL of sample cDNA and 6 µL nuclease-free water. For each PCR reaction, every sample had two technical replicates and two negative controls. Cyclophilin was the housekeeping gene that was used to normalize the expression of the cytokines analysed, applying the 2^−ΔΔCt^ method (relative quantification). Table 3 lists the sequence of the forward and reverse primers (Sigma-Aldrich, Darmstadt, Germany) used alongside with its amplicon length. Table 4 shows the distinct thermal cycle parameters applied for each cytokine analysed, PCR efficiency and R values obtained.

**Table 3:** **Details of primer sequences for RT-PCR assessment of pulmonary cytokines in pigs challenged with *S. Choleraesuis***

Target	Amplicon size (base pairs)	Primer Forward (5ʹ–3ʹ)	Primer Reverse (5ʹ–3ʹ)	References
*TNFα*	134	ACTCGGAACCTCATGGACAG	AGGGGTGAGTCAGTGTGACC	[76]
*IFNγ*	79	TGGTAGCTCTGGGAAACTGAATG	GGCTTTGCGCTGGATCTG	[76]
*CCL28*	145	GCTGCTGCACTGAGGTTTC	TGAGGGCTGACACAGATTC	[68]
*IL-1α*	274	GCTGCTGTGCTAAATAACCT	CTTGTGGCAATAAACAACTTT	[77]
*IL-8*	240	TCTTGGCAGTTTTCCTGCTT	CAACCTTCTGCACCCACTTT	[77]
*Cyclophilin*	369	TAACCCCACCGTCTTCTT	TGCCATCCAACCACTCAG	[69]

CCL28, C–C Motif chemokine ligand 28; IFNγ, Interferon gamma; IL-1α, Interleukin 1 alfa; IL-8, Interleukin 8; TNFα, Tumour necrosis factor alpha.

**Table 4:** **Quantitative PCR conditions and efficiency, R values and number of cycles used for each porcine cytokine and cyclophilin cDNA**

PCR conditions and efficiency							
Cytokine	Initial Denaturation	Denaturation	Annealing	Elongation	Number of PCR cycles	PCR efficiency	R
TNFα	95 °C (10 min)	95 °C (15 s)	60 °C (1 min)		40	2.168	0.994
IFNγ	95 °C (10 min)	95 °C (15 s)	60 °C (1 min)		40	1.982	0.999
CCL28	95 °C (3 min)	95 °C (15 s)	56 °C (30 s)	60 °C (30 s)	45	2.097	0.997
IL-1α	94 °C (2 min)	94 °C (20 s)	60 °C (1 min)		45	1.983	0.999
IL-8	94 °C (2 min)	94 °C (20 s)	60 °C (1 min)		45	1.992	0.998
Cyclophilin	94 °C (3 min)	94 °C (45 s)	54 °C (45 s)	60 °C (45 s)	30	1.864	0.999

CCL28, C–C Motif chemokine ligand 28; IFNγ, Interferon gamma; IL-1α, Interleukin 1 alfa; IL-8, Interleukin 8; TNFα, Tumour necrosis factor alpha.

### Statistical analysis

Data analysis was performed in R 4.0.4 using a fit linear mixed-effects model for data collected ante-mortem (clinical signs, temperature increment, body weight gain, C3, serum cytokines, oxidative stress biomarkers and faecal shedding) and the non-parametric test Mann–Whitney–Wilcoxon for post-mortem data (bacterial tissue colonization, macroscopic and histopathological lesions and cytokine expression in the lung). To reduce skewness of the original data, a logarithmic transformation was applied to faecal shedding and bacterial tissue colonization. For the fit linear mixed-effects model [outcome variable ~ group * day + (1 | individual)] each ante-mortem data was used as the outcome variable, fixed factors were “group” and “day” (categorical variable), and “individual” was included as a random factor. The estimation method used in this case was the Restricted Maximum Likelihood (REML) and, to prove model fit, the residual plot was analysed to ensure lack of patterns and normality presence. As for the post-mortem data, only two groups were statistically analysed as the control group served as a reference of the basal level and was not included in this statistical analysis. Correlation between cytokine expression and microscopic lung lesion score was executed via Spearman’s correlation test due to the non-gaussian data distribution used. The data are shown as mean ± standard error of the mean (SE). For all analysis, statistical significance was declared using a *p*-value ≤ 0.05 with a confidence level (CL) of 95%.

## Results

### Oral immunization with HIMB improves weight gain and reduces *S*. Choleraesuis clinical signs in pigs

**Figure 1:**
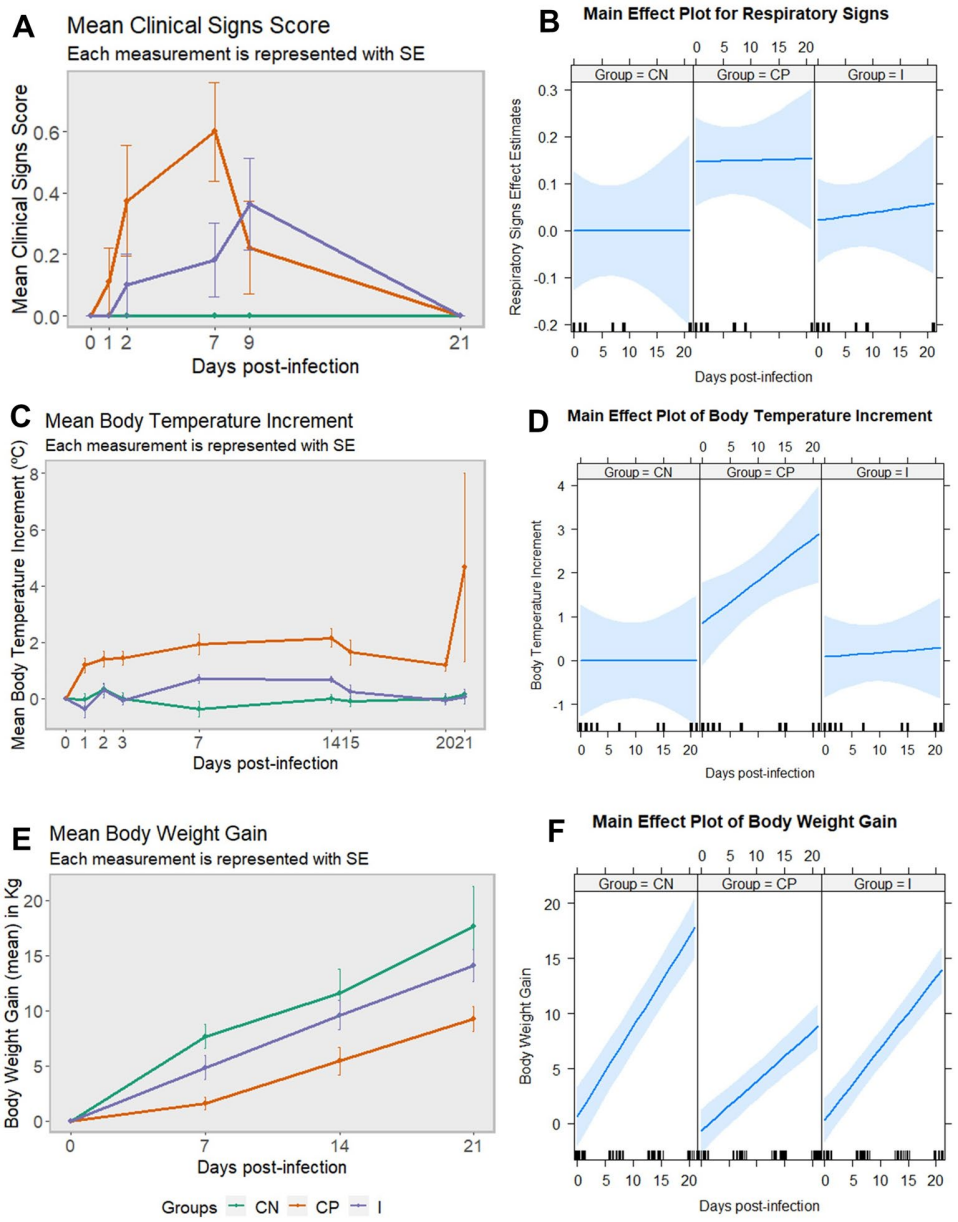
**Clinical signs, temperature increment and weight gain in pigs infected and non-infected with *Salmonella* Choleraesuis.** Evolution of clinical signs score (**A**), temperature increment (**C**) and body weight gain (**E**) per group and days post-infection. Main effect plot for respiratory signs (**B**), temperature increment (**D**) and weight gain (**F**) using the combined effect of fixed variables group and day. CN: negative control group; CP: positive control group; I: immunized group; SE: standard error of the mean.

Clinical signs were mainly respiratory (dyspnoea, cough, sneezing and aphonia) and only individuals from the positive control group presented severe breathing difficulty. Digestive symptoms (diarrhoea) were mild and sporadic in both groups. Apathy and depression were also registered, mostly within the positive control individuals. Only one animal, belonging to the immunized group, had to be euthanized 13 dpi due to septicaemia. During this experiment, a total of 7/9 (77.8%) individuals from the positive control group were symptomatic versus 4/10 (40.0%) of animals from the immunized group. Piglets from the negative control did not show signs of illness. Although non-significant (*p* > 0.05), the mean clinical signs score was higher in the positive control group and appeared earlier than in the immunized group, emphasizing the rapid onset of this disease (Figure 1A). Moreover, respiratory signs were considerably more pronounced in positive controls, with a significant difference at 7 dpi (*p* = 0.0003) (Figure 1B).

Body temperature increment was evident for the positive control group since 1 dpi. Meanwhile, the immunized group maintained its temperature constant, with a slight increase at 7 and 14 dpi. Statistical analysis showed significantly higher temperatures of positive control individuals when compared to immunized ones (*p* = 0.0076) and negative controls (*p* = 0.0125) (Figure 1C). No significant differences were registered between the negative control and the immunized group (*p* > 0.05) (Figure 1D).

There were statistically significant differences in daily weight gain between groups (group*day interaction, *p* = 0.0008). Weight gain was significantly smaller in the positive control group as compared to the negative control (*p* = 0.0041) and immunized (*p* = 0.0441) groups (Figure 1E). A strong effect of group and day variables was evident on weight gain, being higher for negative control and immunized groups (Figure 1F).

### HIMB is not capable of reducing *S*. Choleraesuis shedding or organ dissemination

*S*. Choleraesuis shedding peaked at 14 dpi for the positive control and immunized groups and was also recorded at 21 dpi but with less CFU/gram of faeces (data not shown). Differences in bacterial faecal excretion between groups were non-significant (*p* > 0.05).

Bacterial colonization was especially evident in the tracheobronchial LNs, affecting positive control and immunized groups equally (around 70% of all animals). Bacteria were isolated from palatine tonsils of 66% of the positive control individuals, contrasting with only 24% of the immunized ones. Spleen was the less colonized organ, with three animals (33%) from the positive control group affected and only one individual (12.5%) from the immunized group. Despite these observations, there were no significant differences (*p* > 0.05) between groups for total tissue or individual organ colonization (palatine tonsil, spleen and tracheobronchial LNs). However, a reduction of almost 10^2^ CFU/g between immunized and positive control groups for total tissue bacterial colonization was observed (data not shown).

### Immunostimulation with HIMB reduces *S*. Choleraesuis gross pulmonary lesion score in pigs

After *S*. Choleraesuis challenge, post-mortem examinations revealed that macroscopic lesions were present across 8/9 (88.9%) of individuals from positive control and immunized groups. Control negative animals showed no remarkable gross lesions.

**Figure 2:**
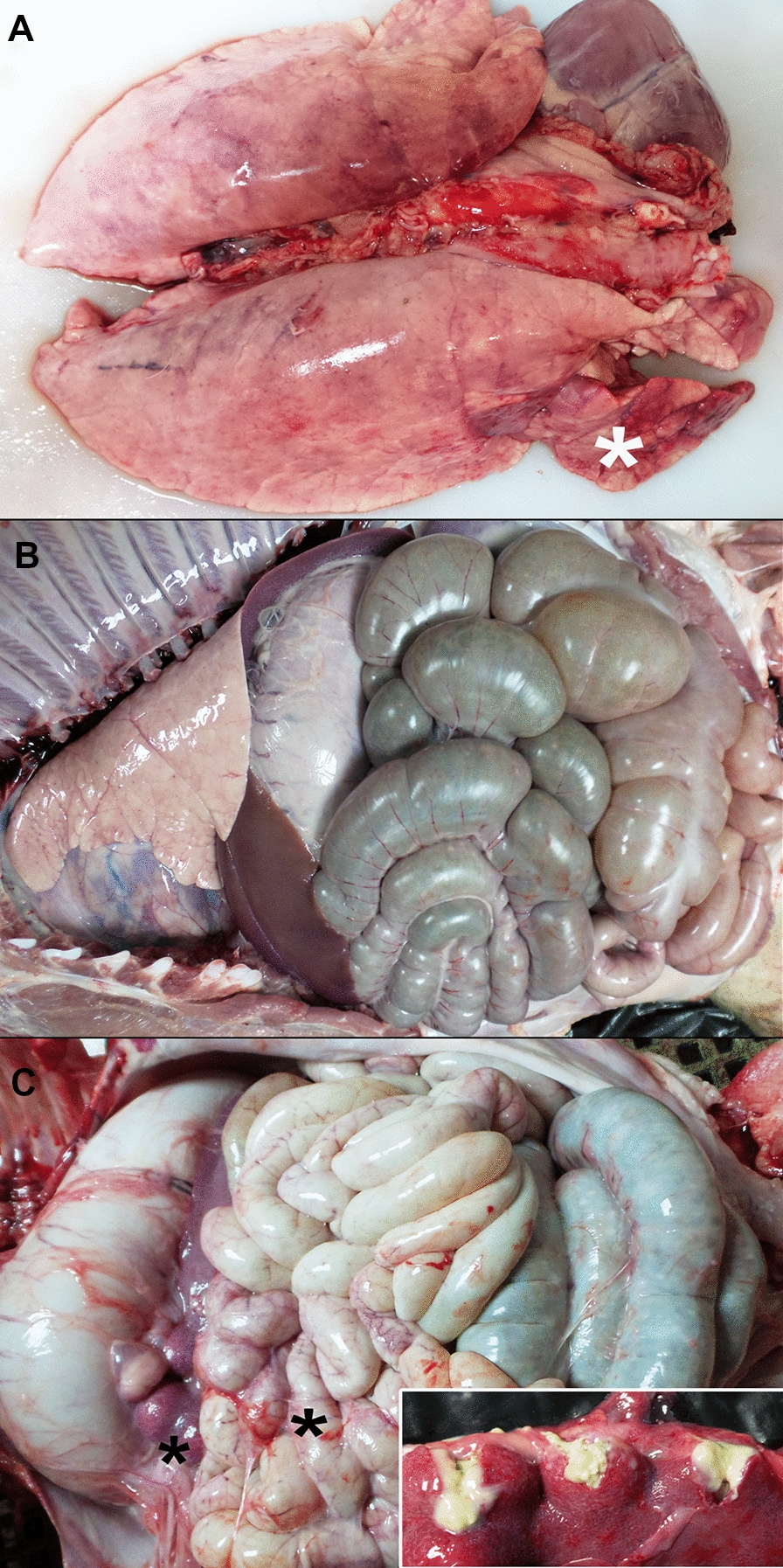
**Macroscopic lesions in pigs infected with *Salmonella* Choleraesuis at 21 days post-infection.** **A** Lung of an animal from the positive control group presenting an increased size and moderate interstitial pneumonia in the right cranial and middle lobes (white asterisk). **B** Thoracic and abdominal cavities of an animal from the immunized group presenting organs without lesions. **C** Spleen and intestine of an animal from the positive control group showing adherence of connective tissue (black asterisks); moreover, the spleen presents multiple abscesses of purulent material (inset).

**Figure 3:**
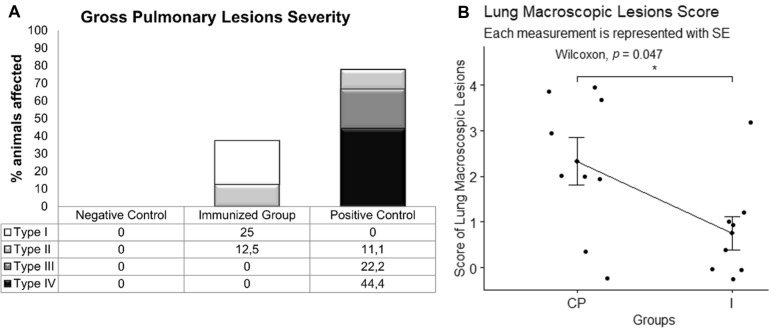
**Characterization of lung macroscopic lesions caused by *Salmonella* Choleraesuis at 21 days post-infection.** **A** Lung gross lesions severity and percentage of piglets affected in each group. **B** Lung macroscopic lesions score separated per groups. CP: positive control group; I: immunized group; SE: standard error of the mean; Type I: interstitial pneumonia with 1 lobe affected; Type II: interstitial pneumonia with 2 lobes affected; Type III: interstitial pneumonia with 3 lobes affected; Type IV: interstitial pneumonia with 4 lobes affected.

The main lesions observed in positive control and immunized groups were interstitial pneumonia, mostly located in the cranial and middle lobes, combined with areas of atelectasis (Figure 2A). In this case, 7/9 (77.8%) of pigs from the positive control group presented gross pulmonary lesions versus 4/9 (44.4%) from the immunized group (Figure 3A). Regarding lesion severity, 44% of the positive control group individuals presented four affected lobes with interstitial pneumonia, while in immunized individuals there were only one (25%) or two lobes (12.5%) with these lesions (Figures 2B and 3A). Furthermore, the lung was one of the most affected organs. Analysing individual pulmonary macroscopic lesions demonstrated that lung lesion scores were higher in the positive control group (*p* = 0.0472) (Figure 3B).

The presence of lesions in the tracheobronchial LNs was observed in 38% of the positive control animals, compared to 75% of the immunized individuals. LNs were mostly hypertrophic and congestive in both groups. Hydropericarditis was registered in 38% of the positive control animals and in 13% of those from the immunized group. Regarding intestinal lesions, 25% of the positive control animals showed enteritis and, as for the immunized group, 38% only presented intestinal vascular changes (congestion or petechial haemorrhages). No signs of enteritis were observed in the immunized group (Figure 2B). Splenomegaly was reported in 25% of the positive control group and 13% of the immunized one. Moreover, one individual from the positive control group presented seven encapsulated splenic abscesses with purulent content (Figure 2C). Lastly, multifocal necrotic hepatic lesions were observed in two of the immunized animals and three liver abscesses were present in one individual from the positive control group.

The analysis of the total macroscopic lesions score revealed no significant differences between immunized and positive control groups (*p* > 0.05), although the total mean score of the immunized group (3.38 ± 0.86) was 42% smaller than the positive control (5.89 ± 0.98) one.

### Oral immunization with HIMB reduces *S*. Choleraesuis histopathological thoracic lesion score in pigs

**Figure 4:**
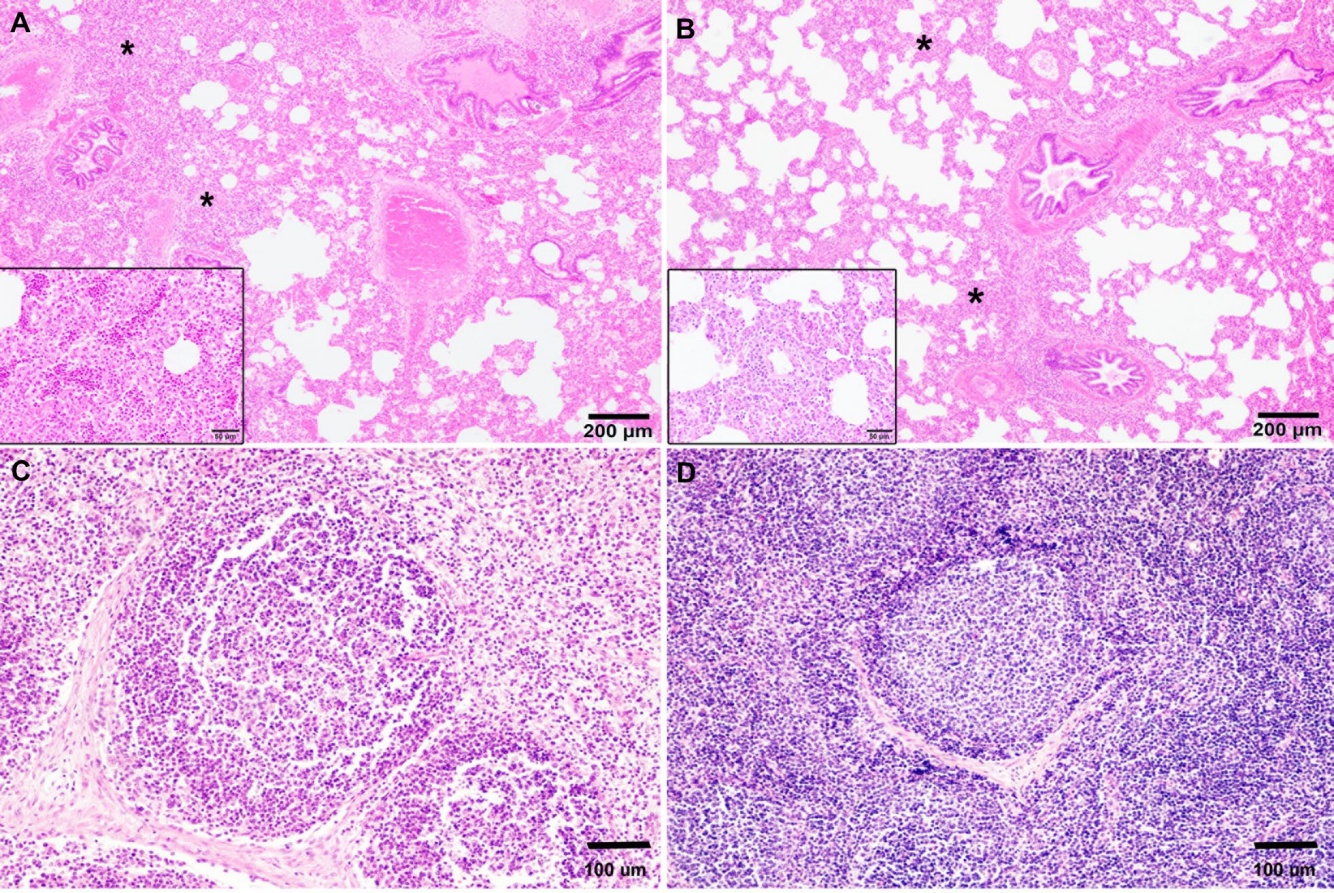
**Histopathological lesions in pigs infected with *Salmonella* Choleraesuis at 21 days post-infection.** **A** Lung of an animal from the positive control group presenting a moderate mixed interstitial inflammatory infiltrate (*), mainly composed of macrophages (MΦs) and lymphocytes (inset). **B** Lung of an animal from the immunized group showing a light thickening of the septa (*), due to infiltrate particularly of MΦs (inset). **C** Tracheobronchial lymphoid node of an animal from the positive control group presenting severe lymphoid depletion and compared with an animal from the immunized group (**D**). Haematoxylin–eosin stain.

**Figure 5:**
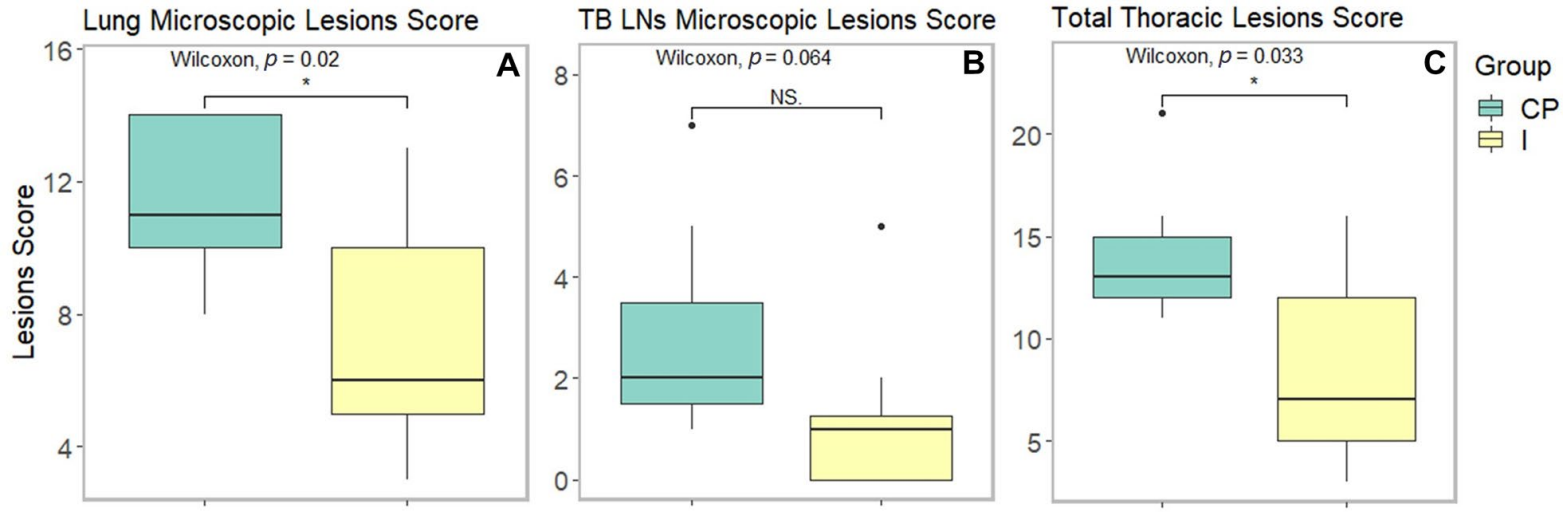
**Microscopic lesions score in pigs infected with *Salmonella* Choleraesuis.** Lung (**A**), tracheobronchial lymph nodes (**B**) and total thoracic (**C**) microscopic lesions score in pigs infected with *Salmonella* Choleraesuis: a comparison between immunized and positive control groups. CP: positive control; I: immunized; LNs: lymph nodes; NS: not significant; TB: tracheobronchial.

For both challenged groups, the main microscopic lesions observed in lung tissue were mononuclear (MΦs and lymphocytes) or mixed (MΦs, lymphocytes, and neutrophils) infiltrates, bronchial epithelial shedding, congestion, interstitial oedema, interstitial haemorrhage and hemosiderosis. Moderate-severe mononuclear infiltrate, mainly constituted by MΦs, was present in 44% of the positive control animals (Figure 4A), while 77% of the individuals from the immunized group presented a mild mononuclear infiltrate (Figure 4B). Only in the positive control group, 44% of individuals presented a mild/moderate alveolar oedema. Liquefactive necrosis was found in two animals from the positive control group and in one individual from the immunized group. The total microscopic lung lesion score was significantly higher (*p* = 0.0204) in the positive control group when compared to the immunized one (Figure 5A).

Tracheobronchial LNs from 75% of the positive control group revealed moderate lymphoid depletion, whilst only one individual from the immunized group had a mild presentation of this lesion (Figures 4C and D). Also, congestion was found in more individuals from the positive control group (57%) than the immunized one (25%). Oedema occurred in two individuals from both groups, while hemosiderosis and liquefactive necrosis were only found in one individual from the immunized group. Differences in total tracheobronchial LN scores between groups were marginally significant (*p* = 0.0639) (Figure 5B), showing a tendency for lesion reduction in the immunized group.

The total thoracic microscopic lesions score (sum of lung and tracheobronchial LNs lesion score) was larger (*p* = 0.0334) in the positive control group when compared to the immunized one (Figure 5C).

### HIMB stimulation lowers MDA levels and enhances SOD activity in pigs infected with *S*. Choleraesuis

**Figure 6:**
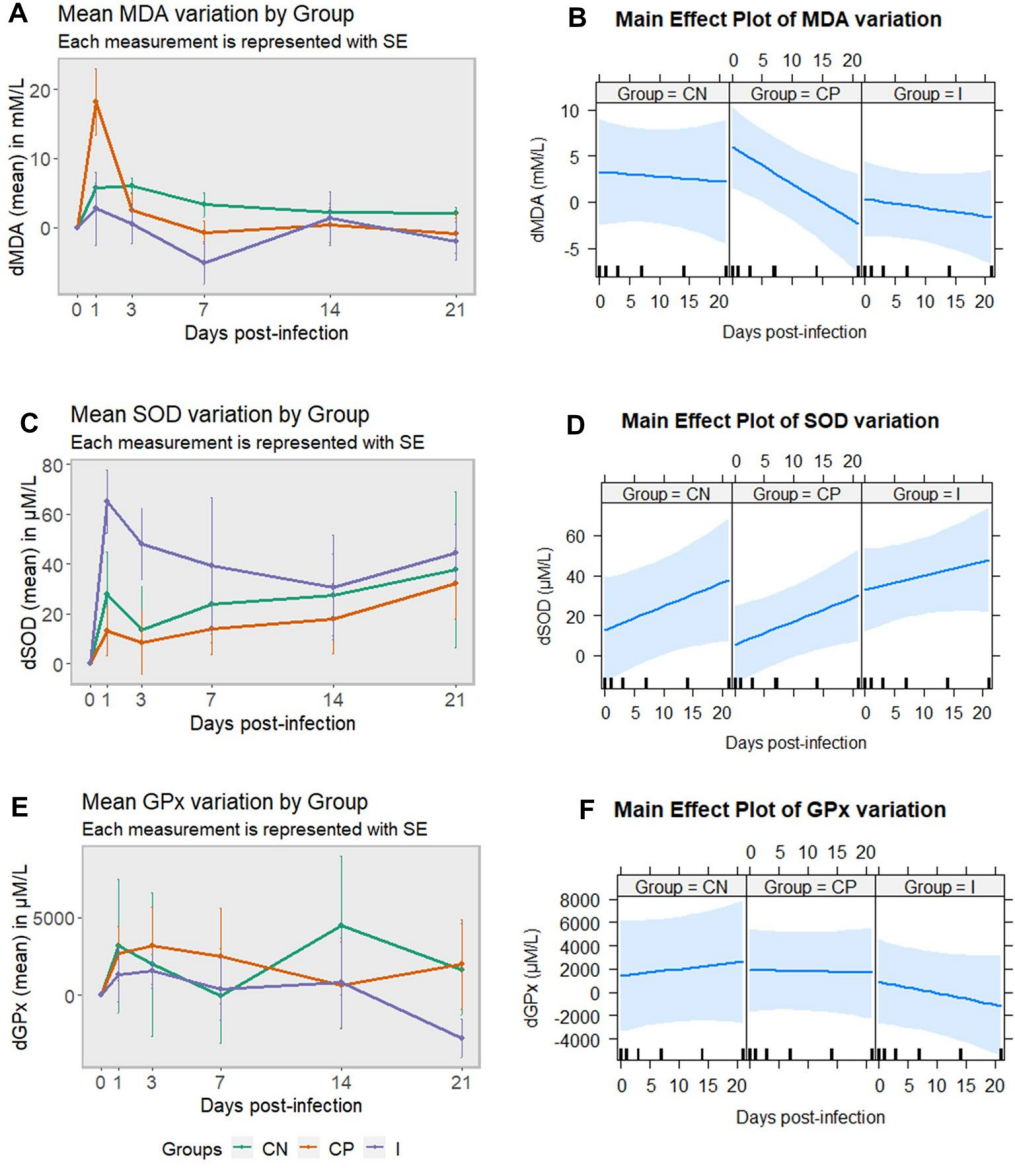
**Oxidative stress biomarkers (dMDA, dGPx and dSOD) in pigs infected and non-infected with *Salmonella* Choleraesuis.** Evolution of the variation in concentration of MDA (**A**), SOD (**C**) and GPx (**E**) per group at different time points, using day 0 as starting point. Main effect plot for MDA (**B**), SOD (**D**) and GPx (**F**) variation, using the combined effect of group and day. CN: negative control group; CP: positive control group; I: immunized group; MDA: malondialdehyde; SOD: superoxide dismutase; GPx: glutathione peroxidase; SE: standard error of the mean.

Plasma MDA variation levels revealed a significant concentration peak 1 dpi for the positive control group compared to the immunized group (*p* = 0.0444) (Figure 6A). This led to a negative slope on the main effect plot for the positive control group (Figure 6B), uncovering the impact that factor group combined with day had on this parameter. By contrast, SOD activity variation of immunized piglets showed an increasing trend from 0 to 1 dpi, maintaining higher values than the other groups until 7 dpi (*p* > 0.05) (Figure 6C). As a result, the positive slope obtained in the main effect plot for all groups presented less steepness in the immunized one (Figure 6D). Blood GPx variation showed no significant activity differences between groups (*p* > 0.05 (Figures 6E and F).

### HIMB triggers an increase in the stimulation of the innate immune component TNFα in pigs infected with *S*. Choleraesuis

Serum C3 variation at different time points revealed no significant concentration changes between groups (*p* > 0.05). Nevertheless, 7 dpi C3 levels were slightly lower in the positive control group (896.71 ± 34.88 µg/mL) when compared to immunized (918.73 ± 9.23 µg/mL) and negative control (927.32 ± 4.68 µg/mL) groups (data not shown).

**Figure 7:**
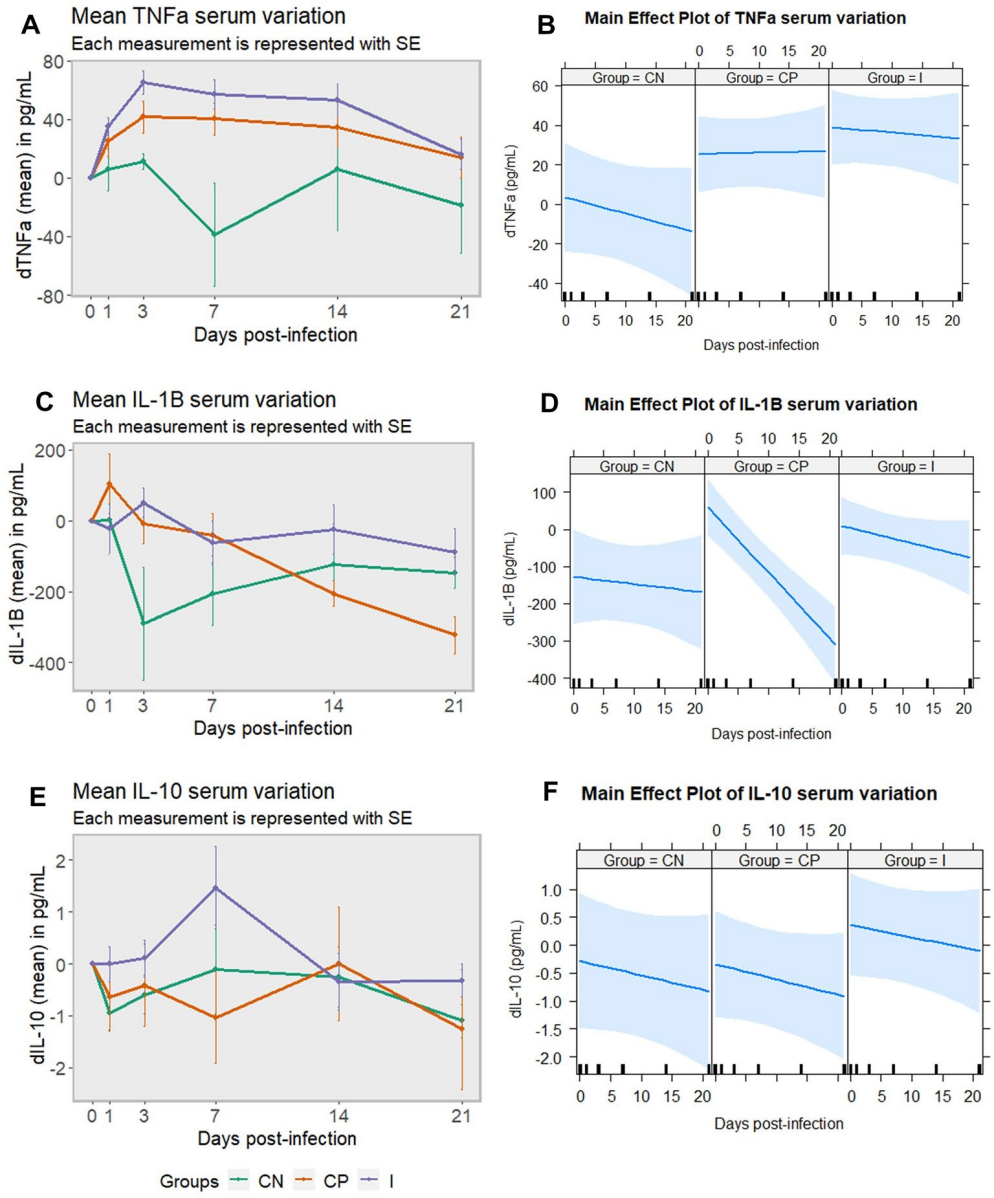
**Cytokine serum concentration (dTNFα, dIL-1β and dIL-10) in pigs infected and non-infected with *Salmonella* Choleraesuis.** Evolution of the variation in concentration of TNFα (**A**), IL-1β (**C**) and IL-10 (**E**) per group at different time points, using day 0 as starting point. Main effect plot for TNFα (B), IL-1β (**D**) and IL-10 (**F**) and variation, using the combined effect of group and day. CN: negative control group; CP: positive control group; I: immunized group; TNFα: tumour necrosis factor α; IL-1β: interleukin 1 beta; IL-10: interleukin 10; SE: standard error of the mean.

TNFα serum variation showed significantly higher concentrations in the immunized group when compared to the negative control one (*p* = 0.0248) (Figure 7A), also evidenced in the main effect plot (Figure 7B). However, although TNFα concentration variation was high in individuals from the control positive group, it was non-significant when compared to the negative one (*p* > 0.05). IL-1β serum concentration variation did not show significant differences among groups (*p* > 0.05) but, while values from immunized and negative control groups remained relatively constant, levels from the positive control group significantly decreased from 1 to 21 dpi (*p* = 0.0003) (Figures 7C and D). The IL-10 serum variation pattern did not present significant differences between groups (*p* > 0.05). Values from the immunized and positive control groups suffered small fluctuations, creating a peak 7 dpi in the immunized group (Figure 7E), leading to higher values for this group in the main effect plot (Figure 7F).

### HIMB does not elicit humoral response against MTC in immunized pigs

Anti-bPPD and anti-P22 antibodies were not detected in the serum of animals from the experimental groups at any time point analyzed throughout this study.

### HIMB immunostimulant increases lung expression of chemokine CCL28 in pigs infected with *S*. Choleraesuis

The housekeeping gene showed uniform expression, with no more than small individual variations in all animals (coefficient of variation [CV] = 5.49). No significant changes (*p* > 0.05) in the immunized group were observed when compared to the positive control group for IFNγ, TNFα, IL-1α and IL-8. Expression of the chemokine CCL28 was significantly higher (*p* = 0.021) in the immunized group when compared to the positive control one. Cytokine gene expression was found to have no significant correlation (*p* > 0.05) with the lung microscopic lesion score. However, there was a tendency for a moderate negative correlation (*p* = 0.1430; R^2^ = 0.32) between lung lesion score and CCL28 expression in the immunized group.

## Discussion

We provide first evidence of a non-specific protective effect of heat-killed mycobacteria in pigs challenged with *Salmonella*. We found that HIMB improved the weight gain and reduced the clinical signs and pulmonary lesions caused by *S.* Choleraesuis in pigs. Pigs stimulated with HIMB showed higher TNFα serum concentration and lung CCL28 expression. Regarding oxidative stress biomarkers, pigs stimulated with HIMB showed lower MDA levels and higher SOD activity than untreated challenged pigs. The excretion and tissue colonization of *S*. Choleraesuis remained however unaffected**.**

An increasing concern from both producers and consumers regarding antibiotic-resistant pathogens dictates the need for new research objectives [48]. The use of vaccines against *Salmonella* might be a suitable alternative in pigs [49]. Nonetheless, the selection of a vaccination protocol to reduce *Salmonella* colonization, excretion and lesions is complicated due to the array of distinct serovars that can be naturally found in swine, as humoral immunity is serogroup specific and cell mediated immunity acts in a non-serogroup specific way [50]. In these cases of low-efficient vaccines, training the innate immunity could be used to optimize the adaptive response to classical vaccines [4, 5]. So, the use of HIMB as an immunostimulant combined with homologous vaccines against this pathogen could eventually improve serovar cross-protection [51].

In our study, HIMB reduced the clinical signs caused by *S*. Choleraesuis, especially breathing difficulty, apathy, and depression. A similar effect was previously observed with other immunostimulants, such as BCG, which was able to increase markedly the resistance of mice to heterologous infection with *Staphylococcus aureus* [52] and *Salmonella* Enteritidis [53]. Our results also showed that HIMB significantly improved weight gain, despite *S*. Choleraesuis challenge, thereby improving productivity and, potentially, reducing economic losses [49]. This effect on weight gain had also been observed when using homologous vaccination against *S*. Choleraesuis [54, 55].

However, HIMB was not capable of reducing *S*. Choleraesuis shedding or organ dissemination. Our observations in relation to *S*. Choleraesuis faecal shedding showed a peak at 14 dpi and continued shedding until 21 dpi in both infected groups, producing similar levels of bacterial environmental contamination. In contrast to our findings, piglets vaccinated intramuscularly with inactivated *S*. Choleraesuis and challenged with a virulent strain did not present environmental shedding [54]. Therefore, aiming a risk mitigation strategy in pig farms, homologous protection seems to have more effective results than heterologous stimulation regarding bacterial shedding [39]. Nevertheless, HIMB showed a tendency to reduce organ colonization of *S.* Choleraesuis with a particular reduction of tissue dissemination on tonsils. This is relevant since this organ has been implicated in the carrier status of pigs infected by *Salmonella* [56]. Likewise, *S*. Choleraesuis-challenge priming with *S*. Typhimurium and boosted with oral inactivated *S*. Choleraesuis was also able to limit organ colonization and more effectively than prime-boost vaccination with homologous vaccine [54].

The thoracic organs were the most affected ones in challenged pigs, both HIMB-treated and untreated, where interstitial pneumonia was the predominant lesion in the lung. Besides, tracheobronchial LNs presented hypertrophy, congestion, and lymphoid depletion. These pathological findings are compatible with *S.* Choleraesuis infections in swine [37, 42, 43]. However, in contrast to these studies, no pulmonary abscesses or intestinal button-shaped ulcers were found, probably because most animals did not show signs of septicaemic disease [44]. In our experiment, immunostimulation with HIMB reduced gross and histopathological lung lesions and caused fewer tracheobronchial LN lesions, particularly lymphoid depletion, in pigs challenged with *S*. Choleraesuis. Similar results were produced by a PCV2 immunostimulant in *S*. Choleraesuis challenged pigs when pulmonary and ileocolic LNs were analysed [57]. In contrast, homologous protection with a live attenuated vaccine against *S*. Choleraesuis did not reduce lesions in challenged pigs [55].

In this experiment, *Salmonella* infection increased MDA levels and reduced antioxidant enzymes in the non-immunized animals, as previously described by Shukla et al. [58]. However, the use of HIMB lowered MDA levels and raised SOD activity, leading to an attenuation of the oxidative stress induced by *S*. Choleraesuis and, consequently, reduced tissue damage [59]. MDA epitopes have been reported to trigger the innate immune response [60] and another study unravels that antioxidant enzymes are involved in the induction of trained immunity [61]. Moreover, an experiment using probiotic *Bacillus* species as an immunomodulator in *S*. Typhimurium challenged mice did not affect MDA concentrations but enhanced total antioxidant capacity [62]. Our study contributes to preliminary data on the effect of mycobacteria-derived immunostimulants on oxidative stress biomarkers using a heterologous challenge.

Pro-inflammatory cytokines like TNFα and IL-1β, and anti-inflammatory IL-10 play an important role in the pathophysiological processes occurring in porcine salmonellosis caused by *S*. Choleraesuis [40, 63, 64]. Our results showed that HIMB immunization gave rise to higher TNFα serum concentration and maintenance of IL-1β values. Previously it had been reported that lactic acid bacteria probiotics increased TNFα serum levels [65]. A similar outcome had also been described in vitro by Kleinnijenhuis et al. [11] with BCG immunostimulant in response to heterologous bacterial and fungal agents. All these findings reinforce the existence of a certain heterologous protection elicited by these immunomodulators. Furthermore, the use of HIMB allowed the maintenance of C3 serum levels, corroborating previous results of HIMB-induced trained immunity involvement [25–27]. With respect to the immunological mechanisms implicated in this non-specific protection against pulmonary lesions, HIMB immunostimulant increased the expression of CCL28 in lung. CCL28 is a chemokine expressed in the lung mucosal tissue and takes an important part in the defence against *Salmonella* [66, 67], playing also an important role in the trained immune protection of the epithelial surfaces [68]. This non-specific immune mechanism of protection is more significant in the lung, since the aggregations of lymphoid tissue are scarce, restricting cellular immune components available [69].

Immunization with HIMB via the oral route did not induce the production of specific anti-MTC antibodies in pigs, responding in a very similar way to that observed in previous studies, as in orally HIMB or BCG vaccinated badgers [17], cattle [70, 71], cervids [72] and wild boars [19], which showed a lack of cellular and humoral response to oral immunization. However, in these studies the level of immunological response increased after *M. bovis* infection, more than in the non-immunized group, with vaccination inducing a reduction of disease severity. In our study, the response of anti-MTC antibodies was evaluated until less of 65 days post-immunization and it is possible that induction of systemic immunity following oral vaccination with HIMB may be delayed, as previously observed following oral BCG vaccination [70]. The absence of sensitization after oral immunization avoids the possibility to develop false-positive reactions in tuberculosis diagnostic tests [72].

This study has some limitations for the interpretation of results. The relatively small number of individuals involved in this experiment may not have sufficient statistical power, causing some findings to show only a tendency rather than a significant result. The 10^6^ CFU intratracheal challenge, used to ensure that all animals received the same dose and guaranteed infection [73, 74], may not fully mimic the natural way of exposure that other studies tried to recreate using the oral challenge route [54, 55] or the intranasal one [75]. This leaves the possibility that immune checkpoints of the upper respiratory tract, potentially stimulated by immunization, could have been bypassed [25, 76]. Thus, the challenge protocol used in this study may have led us to underestimate the protection conferred by HIMB immunostimulation, which might be greater under field conditions with natural exposure or experimentally when mimicking the natural route of infection.

In addition, this study provides interesting insights as it paves the way for immunostimulant development, due to the capacity of HIMB to promote non-specific stimulation of keystone elements related to trained immunity. Training the innate immune cells generates clinical and pathological benefits against heterologous pathogens thereby possibly contributing to fill the gap that traditional vaccines present.

To conclude, we provide first evidence that training the pig immune response with HIMB reduced clinical signs and limited weight loss, thus improving animal welfare and reducing economic losses to pig farming due to *S.* Choleraesuis infections. Animal health status was also improved due to reduced lung lesion scores in HIMB immunostimulated pigs, which can be linked to the reduction of oxidative stress, TNFα triggering and lung CCL28 overexpression, signalling the importance of innate immunity in the non-specific protection against *S*. Choleraesuis. However, HIMB immunization did not reduce *S*. Choleraesuis excretion and tissue colonization. This proof-of-concept study suggests beneficial clinical, pathological, and heterologous immunological effects against bacterial pathogens within the concept of trained immunity, opening avenues for further research.

## Data Availability

The datasets used and/or analysed during the current study are available from the corresponding author on reasonable request.
